# Reinterpretation of the results of a pooled analysis of dietary carotenoid intake and breast cancer risk by using the interval collapsing method

**DOI:** 10.4178/epih.e2016024

**Published:** 2016-06-02

**Authors:** Jong-Myon Bae

**Affiliations:** Department of Preventive Medicine, Jeju National University School of Medicine, Jeju, Korea

**Keywords:** Breast neoplasms, Risk factors, Carotenoids, Meta-analysis

## Abstract

**OBJECTIVES:**

A pooled analysis of 18 prospective cohort studies reported in 2012 for evaluating carotenoid intakes and breast cancer risk defined by estrogen receptor (ER) and progesterone receptor (PR) statuses by using the “highest versus lowest intake” method (HLM). By applying the interval collapsing method (ICM) to maximize the use of the estimated information, we reevaluated the results of the previous analysis in order to reinterpret the inferences made.

**METHODS:**

In order to estimate the summary effect size (sES) and its 95% confidence interval (CI), meta-analyses with the random-effects model were conducted for adjusted relative risks and their 95% CI from the second to the fifth interval according to five kinds of carotenoids and ER/PR status.

**RESULTS:**

The following new findings were identified: α-Carotene and β-cryptoxanthin have protective effects on overall breast cancer. All five kinds of carotenoids showed protective effects on ER− breast cancer. β-Carotene level increased the risk of ER+ or ER+/PR+ breast cancer. α-Carotene, β-carotene, lutein/zeaxanthin, and lycopene showed a protective effect on ER−/PR+ or ER−/PR− breast cancer.

**CONCLUSIONS:**

The new facts support the hypothesis that carotenoids that show anticancer effects with anti-oxygen function might reduce the risk of ER− breast cancer. Based on the new facts, the modification of the effects of α-carotene, β-carotene, and β-cryptoxanthin should be evaluated according to PR and ER statuses.

## INTRODUCTION

Although many studies have investigated whether carotenoids, which can be mainly consumed from fruits and vegetables, have a preventive effect on breast cancer, the results have been inconsistent [1]. The discrepant results are largely because breast cancer is not a single disorder. In other words, breast cancer exhibits various aspects depending on the estrogen receptor (ER) or progesterone receptor (PR) status and menopausal status [2,3]. Given that carotenoids exert anticancer effects by acting as antioxidants [4], they can be expected to suppress the risk of developing ER-negative (ER−) breast cancer [2,3]. That is, as ER− breast cancer develops regardless of ER expression, the suppressive effect of carotenoids on cancer risk should be more pronounced [4,5]. To investigate this hypothesis, Zhang et al. [5] conducted a pooled analysis by compiling the findings of 18 previous cohort studies into a large database. Such analysis can allow observational studies to deduce results with the highest levels of evidence.

The authors of the study divided carotenoids into five types as follows: α-carotene (AC), β-carotene (BC), β-cryptoxanthin (CX), lutein/zeaxanthin (LZ), and lycopene (LY). They concluded that only AC, BC, and LZ prevented ER− breast cancer. However, Zhang et al. [5] divided carotenoid intakes into five quintiles and deduced this conclusion based only on the relative risk (RR) of the fifth interval, which represented the highest intake of each carotenoid, and its 95% confidence interval (CI). In other words, the “highest vs. lowest intake” method (HLM) was applied [6]. But the HLM does not maximize the use of the given information. Thus, recently, the interval collapsing method (ICM), which can increase the statistical power of the test by using all information from the second to the fifth interval, was suggested [6].

This study aimed to deduce new conclusions by applying the ICM to the study results of Zhang et al. [5], who deduced the aforementioned conclusion by using the HLM. If new findings emerge, then the interpretation of the results will change accordingly and a new hypothesis can be proposed.

## MATERIALS AND METHODS

The ICM was applied on the data provided by Zhang et al. [5] in their Tables 2 and 3, which were the adjusted RR (aRR) and its 95% CI, presented for each of the five intake intervals in each group classified based on the five types of carotenoids, and the ER and PR status. In the ICM, the four aRR values from the second to the fifth interval were natural-log transformed, the reciprocal of the standard error for each interval was calculated, and a meta-analysis was conducted by using a randomeffects model (REM) [6]. This is similar to the procedure to calculate the summary effect size (sES) that was used in the systemic reviews reported in four articles selected via literature search and meta-analyzed. As a result, five sESs and their corresponding 95% CIs were calculated and presented in up to three decimal places. In calculating the sES in the second to the fifth interval, heterogeneity was confirmed based on the I^2^ values (%). The statistical significance level was set at 5%, and Stata version 14.0 (StataCorp, College Station, TX, USA) was used.

## RESULTS

Tables 1 and 2 present the results obtained by applying the ICM to the data presented by Zhang et al. [5] in their Tables 2 and 3, respectively. In comparing the CI in the fifth interval, the CI from the ICM was confirmed to be narrower. The I^2^ values that indicated heterogeneity were all 0.0% because the information from one article was combined.

Table 3 shows the differences in the conclusions deduced from the HLM and those deduced from the ICM. The following interpretations were newly added to the conclusion of Zhang et al. [5]: (1) AC and CX have a protective effect against all breast cancers regardless of ER/PR status; (2) all five types of carotenoids are effective in suppressing ER− breast cancer; (3) BC increases the risk of developing ER+ or ER+/PR+ breast cancer; (4) along with AC, BC, and LY, LZ also suppresses ER−/PR+ breast cancer; and (5) AC, LZ, and LY, along with BC, suppress ER−/PR− breast cancer.

## DISCUSSION

As shown in Table 3, new facts were found by applying the ICM instead of the HLM. First, all the five carotenoid types suppressed ER− breast cancer. This strengthens the hypothesis of Zhang et al. [5] that if the anticancer mechanism of carotenoids is not associated with steroid hormones, then they will have significant suppressive effects on ER− breast cancer. Furthermore, the conclusion that AC, BC, LZ, and LY suppress both ER−/PR+ and ER−/PR− breast cancers represents another new finding that additionally supports the hypothesis of Zhang et al. [5].

By contrast, CX showed protective effects against ER− breast cancer, although its effect showed no statistical significance for ER−/PR+ and ER−/PR− breast cancers (sES, 0.945; 95% CI, 0.896 to 0.997). However, in ER−/PR+ breast cancer, the sES was 0.885, which indicated increased protective effects, though not statistically significant (95% CI, 0.764 to 1.026). In ER−/PR− breast cancer, the sES was 0.968, which demonstrated decreased protective effects, again with no statistical significance (95% CI, 0.916 to 1.022). Although the interaction depending on PR status could have potentially influenced these findings, additional analysis should be conducted by using the original data.

CX was found to have a suppressive effect on the risk of developing all types of breast cancer, regardless of ER or PR status. As CX is closely associated with vitamin C intake [7], additional in-depth analysis of the association between breast cancer risk and vitamin C intake levels is required.

A new finding is that AC, in addition to CX, suppresses all types of breast cancer. Moreover, AC showed an even greater suppressive effect on ER−/PR+ breast cancer (sES, 0.704; 95% CI, 0.614 to 0.808). It also suppressed the development of ER-/PR- breast cancer (sES, 0.913; 95% CI, 0.860 to 0.970), while its CI did not overlap with that for ER−/PR+. This result can mean that the suppressive effects of AC on ER− breast cancer differ according to PR status. This also requires further in-depth analysis.

Noteworthy among the new findings is that BC increased the risk of developing ER+ (sES, 1.037; 95% CI, 1.008 to 1.067) or ER+/PR+ breast cancer (sES, 1.034; 95% CI, 1.005 to 1.065). However, statistical significance was not found for ER+/PR− breast cancer. This can be interpreted to suggest that the suppressive effect of BC on ER+ breast cancer differs according to PR status. This possibility, however, also requires further in-depth analysis. Although all these data require further in-depth analysis, this study has a limitation in that it cannot conduct such analysis because it used only data presented by a previously published study. However, the present study is still significant in that it added new findings to the previous analyses.

In summary, one of the new findings is that all five types of carotenoid suppress ER− breast cancer. Additional analyses are needed because AC, BC, and CX can potentially have varying suppressive effects on breast cancer according to PR status.

## Figures and Tables

**Table 1.** Summary effect sizes with their 95% confidence intervals estimated by using the interval collapsing method^1^

	Overall	ER+	ER-	PR+	PR-
α-carotene	0.978 (0.960, 0.996)^2^	1.020 (0.996, 1.044)	0.895 (0.847, 0.947)^2^	1.001 (0.975, 1.027)	0.979 (0.939, 1.019)
β-carotene	1.007 (0.984, 1.030)	1.037 (1.008, 1.067)^2^	0.893 (0.849, 0.939)^2^	1.017 (0.986, 1.049)	0.970 (0.933, 1.008)	
β-cryptoxanthin	0.974 (0.954, 0.995)^2^	0.984 (0.961, 1.007)	0.945 (0.896, 0.997)^2^	0.979 (0.952, 1.006)	0.967 (0.992, 1.013)	
Lutein/zeaxanthin	1.002 (0.982, 1.023)	1.029 (0.999, 1.059)	0.901 (0.859, 0.945)^2^	1.013 (0.981, 1.047)	0.978 (0.940, 1.019)	
Lycopene	0.990 (0.972, 1.008)	0.998 (0.973, 1.024)	0.933 (0.889, 0.979)^2^	1.001 (0.969, 1.033)	0.952 (0.915, 0.989)^2^	

ER, estrogen receptor; PR, progesterone receptor.

1 From the results presented in Table 2 in Zhang et al. [5].

2 The data are new findings that show statistical significance.

**Table 2.** Summary effect sizes with their 95% confidence intervals estimated by using the interval collapsing method^1^

	ER+/PR+	ER+/PR-	ER-/PR+	ER-/PR-
α-carotene	1.022 (0.995, 1.049)	1.060 (0.999, 1.124)	0.704 (0.614, 0.808)^2^	0.913 (0.860, 0.970)^2^
β-carotene	1.034 (1.005, 1.065)^2^	1.050 (0.990, 1.114)	0.791 (0.686, 0.911)^2^	0.910 (0.862, 0.961)^2^
β-cryptoxanthin	0.982 (0.958, 1.007)	0.974 (0.912, 1.040)	0.885 (0.764, 1.026)	0.968 (0.916, 1.022)
Lutein/zeaxanthin	1.018 (0.998, 1.049)	1.057 (0.993, 1.125)	0.753 (0.650, 0.873)^2^	0.907 (0.860, 0.956)^2^
Lycopene	1.008 (0.975, 1.042)	0.952 (0.893, 1.016)	0.802 (0.684, 0.941)^2^	0.932 (0.884, 0.983)^2^

ER, estrogen receptor; PR, progesterone receptor.

1 From the results presented in Table 3 in Zhang et al. [5].

2 The data are the new findings that show statistical significance.

**Table 3.** Comparison of the carotenoids that show statistical significance between the HLM and ICM

	HLM	ICM
Overall	CX	+AC
ER-	AC, BC, LZ	+CX, LY
ER+	none	+BC (risky)
ER+PR+	none	+BC (risky)
ER+/PR-	none	none
ER-/PR+	AC, BC, LY	+LZ
ER-/PR-	BC	+ AC, LZ, LY

HLM, highest versus lowest intake method; ICM, interval collapsing method; ER, estrogen receptor; RR, progesterone receptor; AC, α-carotene; BC, β-carotene; CX, β-cryptoxanthin; LZ, lutein/zeaxanthin; LY, lycopene.
