# Supplementary material for: Reinterpretation of the results of a pooled analysis of dietary carotenoid intake and breast cancer risk by using the interval collapsing method
Source: Epidemiol Health. 2016 Jun 2;38:e2016024. doi: 10.4178/epih.e2016024 (PMC4974449; doi:10.4178/epih.e2016024)
Supplement: Supplementary file 1 [file epih-38-e2016024-supplementary.pdf]

<Brief Communication>

Reinterpretation of the Results of a Pooled Analysis of Dietary Carotenoid Intake and Breast Cancer Risk by Using the Interval Collapsing Method

Jong-Myon Bae

Department of Preventive Medicine,

Jeju National University School of Medicine

Corresponding authors: Jong-Myon Bae

jmbae@jejunu.ac.kr

064-755-5567

서론

그동안 과일과 채소로 섭취하는 Carotenoids 가 유방암의 예방 효과가 있는가를 규명하는 수많은 연구들이 있었지만, 그 결과들은 일관성을 보이지 않았다 [1]. 이는 유방암이 단일 질병이 아니기 때문이다. 즉 여성호르몬 수용체 (estrogen receptor, ER; progesterone receptor, PR) 여부, 폐경 여부 등에 따라 다른 양상을 보이기 때문이다 [2,3]. Carotenoids가 항산화제 역할을 하여 항암효과를 갖는다고 볼 때 [4], 여성호르몬 수용체가 없는 ER- 유방암에 있어 특히 발생을 억제할 것으로 예상할 수 있다 [2,3]. 다시 말해서 ER- 유방암은 여성호르몬에 무관하게 발생하는 것이므로, Carotenoids의 암발생억제 효과가 보다 분명할 것으로 예상할 수 있다 [4,5]. 이를 규명하기 위하여 Zhang et al. [5]은 기존의 구축된 18개의 코호트 들을 한 곳에 모아서 하나의 거대한 코호트 데이터베이스를 구축하여 pooled analysis를 수행하였다. 이는 관찰연구 중 가장 근거수준이 높은 결과를 도출할 수 있기 때문이다.

저자들은 산출한 연구결과를 바탕으로 carotenoids를 α-Carotene (AC), β-Carotene (BC), β-Cryptoxanthin (CX), Lutein/Zeaxanthin (LZ), Lycopene (LY)의 5 종류로 나누었을 때 이중 AC, BC, LZ 만이 ER 음성 (ER-) 유방암을 예방한다고 결론지었다. 그런데 이러한 결론 도출은 carotenoids 종류별로 5 분위 섭취수준으로 나눈 뒤, 가장 높은 수준인 제 5 구간의 relative risk (RR)과 이의 95% confidence interval (CI)을 근거한 것이었다. 즉 highest versus lowest intake method (HLM)를 적용한 것이다 [6]. 그런데 HLM은 얻어낸 정보들을 최대한 활용하지 못하는 단점 때문에 2-5 구간 모두의 정보를 활용하여 통계 검정력을 높일 수 있는 interval collapsing method (ICM) 이 제시되었다 [6].

본 연구는 HLM법을 적용하여 결론을 도출한 Zhang et al. [5]의 연구 결과에 대하여, ICM을 적용하여 새로운 결론들을 탐색하고자 한다. 만약 새로운 사실이 도출될 경우 결과의 해석 또한 달라질 것이며, 새로운 가설을 제시할 수 있기 때문이다.

연구 대상 및 방법

ICM 법을 적용할 정보는 Zhang et al. [5] 의 표 2, 3에 제시한 수치들이다. 즉, 5 종류의 carotenoid 와 ER, PR 여부로 나눈 군에 있어 5분위 섭취 수준별로 제시한 adjusted RR (aRR)과 이의 95% CI 이다. ICM 법은 2-5분위의 4가지 aRR을 자연로그화 변환하고, 분위별 표준오차의 역수값을 확보한 다음, random effect model (REM)의 메타분석을 수행하는 것이다 [6]. 마치 체계적 고찰에서 검색으로 선정한 4개 논문을 메타분석하여 summary effect size (sES)를 구하는 것과 같은 의미이다. 그 결과 5 종류별 sES와 이의 95% CI을 산출하였고, 소수점 셋째 자리까지 제시하였다. 그리고 2-5분위의 sES 산출에 있어 이질성 여부 확인은 I-squared 값(%)을 기준으로 평가하였다. 통계적 유의수준을 5%로 정했으며, STATA version 14 (www.stata.com) 통계프로그램을 이용하였다.

연구결과

Table 1과 2는 Zhang et al. [5] 의 표 2, 3에 제시한 수치에 ICM을 적용한 결과들을 각각 제시한 표이다. 제 5 구간의 신뢰구간과 비교할 때, ICM 적용한 신뢰구간은 더 좁혀진 것을 확인할 수 있었다. 한편, 한 논문내의 정보들을 합성하였기에 이질성을 나타내는 I-squared 값은 모두 0.0% 이었다.

Table 3은 HLM법을 적용한 것에 비하여 ICM을 적용하였을 때의 결론 도출 차이를 표로 정리한 것이다. Zhang et al. [5]의 결론에 비하여 다음의 해석들이 새로이 추가되었다. (1) ER/PR을 구분하지 않은 전체 대상에 있어 CX 뿐만 아니라 AC도 보호효과가 있다. (2) ER-유방암 발생에 있어 5 종류 모두 억제 효과가 있다. (3) ER+ 혹은 ER+PR+ 유방암에 있어 BC는 암발생 위험도를 높인다. (4) ER-PR+ 유방암에 있어 AC, BC, LY 뿐만 아니라 LZ도 억제효과가 있다. (5) ER-PR- 유방암에 있어 BC와 함께 AC, LZ, LY 도 보호효과가 있다.

토론

표 3에서 보듯이 HLM에 비하여 ICM을 적용함으로써, 새로운 사실들을 알아낼 수 있었다. 우선 주목할 점은 5 종류의 carotenoids 모두 ER- 유방암 억제한다는 것이다. 이는 carotenoids의 항암기전이 스테로이드 호르몬과 무관하다면 ER- 유방암에서 유의미한 효과를 가질 것이라는 Zhang et al. [5] 의 가설을 더욱 지지하는 소견이기 때문이다. 그리고 ER-PR+ 와 ER-PR- 유방암 모두에서 AC, BC, LZ, LY 가 억제효과가 있다는 같은 결론도 저자들의 가설을 추가로 지지해 주는 새로운 사실이다.

반면 ER-PR+, ER-PR-에서 통계적 유의성을 보이지 않은 CX는 ER- 군에서는 보호효과를 보였다 (sES=0.945, 95% CI: 0.896-0.997). 그러나 ER-PR+ 군에서는 sES가 0.885로 보호효과가 더 커졌지만 통계적 유의성이 사라졌다 (95% CI: 0.764-1.026). ER-PR-군에서는 sES가 0.968 보호효과가 더 줄어들면서 통계적 유의성도 사라졌다 (95%CI: 0.916-1.022). 이런 현상은 PR 여부에 따른 상호작용 (interaction)이 개입될 가능성을 의심할 수 있지만, 원자료에서 추가 분석을 할 필요가 있다.

그런데 CX는 ER, PR과 무관한 전체 유방암의 발생을 억제하는 것으로 나왔다. CX가 vitamin C 섭취와 밀접한 관련이 있다는 점에서 [7], 향후 Vitamin C 수준에 따른 유방암 발생 관련성을 심도있게 분석할 필요가 있겠다.

CX뿐만 아니라 AC도 전체 유방암의 발생을 억제한다는 새로운 사실을 알아내었다. 이에 더하여 AC는 ER-PR+ 유방암에 있어 더 큰 억제효과를 보였다 (sES=0.704, 95% CI: 0.614-0.808). 그리고 EP-PR- 유방암에 있어 억제효과가 여전히 있지만 ER-PR+의 신뢰구간과 겹치지 않는다 (sES=0.913, 95% CI: 0.860-0.970). 이 점은 AC가 ER- 유방암에 있어서 PR 여부에 따라 억제 효과가 다르다고 유추해 볼 수 있다. 이 또한 심층 분석이 필요하다.

새로이 알게 된 사실 중 특이한 것으로, BC가 ER+ (sES=1.037, 95% CI: 1.008-1.067) 또는 ER+PR+ (sES=1.034, 95% CI: 1.005-1.065) 유방암의 발생위험을 높인다는 점이다. 그러나 ER+PR-인 유방암에서는 통계적 유의성이 사라졌다. 이 점은 BC가 ER+ 유방암에 있어서 PR 여부에 따라 작용 효과가 다르다고 유추할 수 있으며, 이 또한 심층 분석이 필요하다. 그러나 이상의 심층 분석들이 필요한 것들이 있음에도, 본 연구에서 이를 수행할 수 없다는 점이 본 연구의 한계이다. 기 발표된 논문에서 제시한 정보만을 이용하였기 때문이다. 다만 기존의 해석에서 새로운 사실들을 추가했다는 점에서 본 연구는 의의를 가질 수 있다.

요약한다면, 그 사실들 중에는 5 종류의 carotenoid는 ER- 유방암 발생을 모두 억제한다는 점이다. 추가로 AC, BC, CX는 PR 여부에 따라 암발생 억제의 효과가 달라질 수 있다는 개연성에 따라 추가적인 분석이 필요하겠다.

Acknowledgement

This study was supported by a grant from the Korean Foundation for Cancer Research, Seoul, Republic of Korea (2013-2).

References

1. World Cancer Research Fund and American Institute for Cancer Research Expert Panel. Food, nutrition and the prevention of cancer: a global perspective. Washington, DC: American Institute for Cancer Research; 2007, p. 289-295.
2. Ursin G, Bernstein L, Lord SJ, Karim R, Deapen D, Press MF, et al. Reproductive factors and subtypes of breast cancer defined by hormone receptor and histology. Br J Cancer 2005;93:364-71.
3. Cotterchio M, Kreiger N, Theis B, Sloan M, Bahl S. Hormonal factors and the risk of breast cancer according to estrogen- and progesterone-receptor subgroup. Cancer Epidemiol Biomarkers Prev 2003;12:1053-60.
4. Rao AV, Rao LG. Carotenoids and human health. Pharmacol Res 2007;55:207-16.
5. Zhang X, Spiegelman D, Baglietto L, Bernstein L, Boggs DA, van den Brandt PA, et al. Carotenoid intakes and risk of breast cancer defined by estrogen receptor and progesterone receptor status: a pooled analysis of 18 prospective cohort studies. Am J Clin Nutr 2012;95:713-25.
6. Bae JM. Comparison of methods of extracting information for meta-analysis of observational studies in nutritional epidemiology. Epidemiol Health 2016;38:e2016003.
7. Chug-Ahuja JK, Holden JM, Forman MR, Mangels AR, Beecher GR, Lanza E. The development and application of a carotenoid database for fruits, vegetables, and selected multicomponent foods. J Am Diet Assoc 1993;93:318-23.

국문요약본

Objectives: Carotenoids 가 항암기전이 있다면 steroid hormone에 무관한 estrogen receptor negative (ER-) 혹은 progesterone receptor negative (PR-) 유방암의 발생을 억제할 것으로 예상할 수 있다. 이를 규명하기 위하여 18개의 코호트에 대한 pooled analysis 결과가 2012년도에 발표되었다. 그런데 저자들은 5 종류의 carotenods - α-Carotene (AC), β-Carotene (BC), β-Cryptoxanthin (CX), Lutein/Zeaxanthin (LZ), Lycopene (LY)- 별로 섭취 수준을 5분위로 나눈 뒤, 5번째 구간의 결과만을 근거하여 해석을 하였다 (highest versus lowest intake method, HLM). 이에 산출한 정보들을 최대한 활용하였을 때 (interval collapsing method, ICM) 새로운 사실들이 있는가를 알아보았다.

Methods: 기 발표된 pooled analysis 결과에 있어 5분위로 제시한 adjusted relative risk and its 95% confidence interval (CI)를 random effect model을 적용한 meta-analysis를 수행하여 summary effect size (sES) and its 95% CI을 구하였다.

Results: ICM을 적용한 결과 다음의 사실들을 새로이 알아낼 수 있었다. (1) ER/PR을 구분하지 않은 전체 대상에 있어 CX 뿐만 아니라 AC도 보호효과가 있다. (2) ER- 유방암 발생에 있어 5 종류 모두 억제 효과가 있다. (3) BC는 ER+ 혹은 ER+PR+ 유방암 위험도를 높인다. (4) ER-PR+ 유방암에 있어 AC, BC, LY 뿐만 아니라 LZ도 억제효과가 있다. (5) ER-PR- 유방암에 있어 BC와 함께 AC, LZ, LY 도 보호효과가 있다.

Conclusions: 5 종류의 carotenoids 모두 ER- 유방암 억제한다는 사실은 carotenoids의 항암기전이 스테로이드 호르몬과 무관하다면 ER- 유방암에서 유의미한 효과를 가질 것이라는 가설을 더욱 지지하는 소견이다. 새로이 해석된 사실에 따라 AC, BC, CX는 PR 여부에 따라 암발생 억제의 효과가 달라질 수 있다는 개연성이 제기되므로 향후 추가적인 분석이 필요하겠다.

Keyword: 유방암, 위험요인, 카로테노이드, 메타분석

Table 1. Summary effect sizes with their 95% confidence intervals estimated by using the interval collapsing method from the results presented in Table 2 in Zhang et al. [5]

	**Overall**	**ER+**	**ER−**	**PR+**	**PR−**
α-Carotene	**0.978 (0.960–0.996)**	1.020 (0.996–1.044)	**0.895 (0.847–0.947)**	1.001 (0.975–1.027)	0.979 (0.939–1.019)
β-Carotene	1.007 (0.984–1.030)	**1.037 (1.008–1.067)**	**0.893 (0.849–0.939)**	1.017 (0.986–1.049)	0.970 (0.933–1.008)
β-Cryptoxanthin	**0.974 (0.954–0.995)**	0.984 (0.961–1.007)	**0.945 (0.896–0.997)**	0.979 (0.952–1.006)	0.967 (0.992–1.013)
Lutein/zeaxanthin	1.002 (0.982–1.023)	1.029 (0.999–1.059)	**0.901 (0.859–0.945)**	1.013 (0.981–1.047)	0.978 (0.940–1.019)
Lycopene	0.990 (0.972–1.008)	0.998 (0.973–1.024)	**0.933 (0.889–0.979)**	1.001 (0.969–1.033)	**0.952 (0.915–0.989)**

The data in bold are new findings that show statistical significance.

Table 2. Summary effect sizes with their 95% confidence intervals estimated by using the interval collapsing method from the results presented in Table 3 in Zhang et al. [5]

	**ER+/PR+**	**ER+/PR−**	**ER−/PR+**	**ER−/PR−**
α-Carotene	1.022 (0.995–1.049)	1.060 (0.999–1.124)	**0.704 (0.614–0.808)**	**0.913 (0.860–0.970)**
β-Carotene	**1.034 (1.005–1.065)**	1.050 (0.990–1.114)	**0.791 (0.686–0.911)**	**0.910 (0.862–0.961)**
β-Cryptoxanthin	0.982 (0.958–1.007)	0.974 (0.912–1.040)	0.885 (0.764–1.026)	0.968 (0.916–1.022)
Lutein/zeaxanthin	1.018 (0.998–1.049)	1.057 (0.993–1.125)	**0.753 (0.650–0.873)**	**0.907 (0.860–0.956)**
Lycopene	1.008 (0.975–1.042)	0.952 (0.893–1.016)	**0.802 (0.684–0.941)**	**0.932 (0.884–0.983)**

The data in bold are the new findings that show statistical significance.

Table 3. Comparison of the carotenoids that show statistical significance between the “highest versus lowest intake” method (HLM) and “interval collapsing” method (ICM)

	**HLM**	**ICM**
Overall	CX	+AC
ER-	**AC, BC, LZ**	+CX, LY
ER+	none	+BC (risky)
ER+PR+	none	+BC (risky)
ER+/PR−	none	none
ER−/PR+	AC, BC, LY	+LZ
ER−/PR−	BC	+ AC, LZ, LY

AC (α-carotene), BC (β-carotene), CX (β-cryptoxanthin), LZ (lutein/zeaxanthin), and LY (lycopene)
